# Supplementary material for: MoS2 based 2D material photodetector array with high pixel density
Source: Nanophotonics. 2025 Jun 20;14(23):4197–205. doi: 10.1515/nanoph-2025-0048 (PMC12617695; doi:10.1515/nanoph-2025-0048)
Supplement: Supplementary file 1 — Supplementary Material Details [file j_nanoph-2025-0048_suppl_001.docx]

Supplementary Materials

*MoS2 Based 2D Material Photodetector Array with high Pixel Density*

*Russell L. T. Schwartz^1^, Hao Wang^1^, Chandraman Patil^1^, Martin Thomaschewski^2^, Volker J. Sorger^1^*

*^1^Department of Electrical & Computer Engineering and Florida Semiconductor Institute, University of Florida*

*^2^Thomas J. Watson Laboratory of Applied Physics, California Institute of Technology*

**Contents:**

**S1.** Bending Radius and Strain Calculations

**S2.** Device Variation: Thickness, Resistance, Size & Placement

**S3.** Fabrication Procedures

**S4.** Dark Current Changes under Bias Voltage Tuning

**S1. Bending Radius and Strain Calculations**

The bending radius was calculated assuming a circular bend, where the width and height of the bend were used to calculate the radius, as presented in Fig. S1.

The calculated bending radius and substrate thickness were then used to find the strain induced on the devices; this calculation assumes that the device sit a negligible height above the surface of the substrate. In Eq. 1 epsilon represents the amount of strain induced on the 2DM devices, while tau is half of the thickness of the substrate showing the area where tensile strain is present on the chip, and R represents the bending radius that was previously calculated.


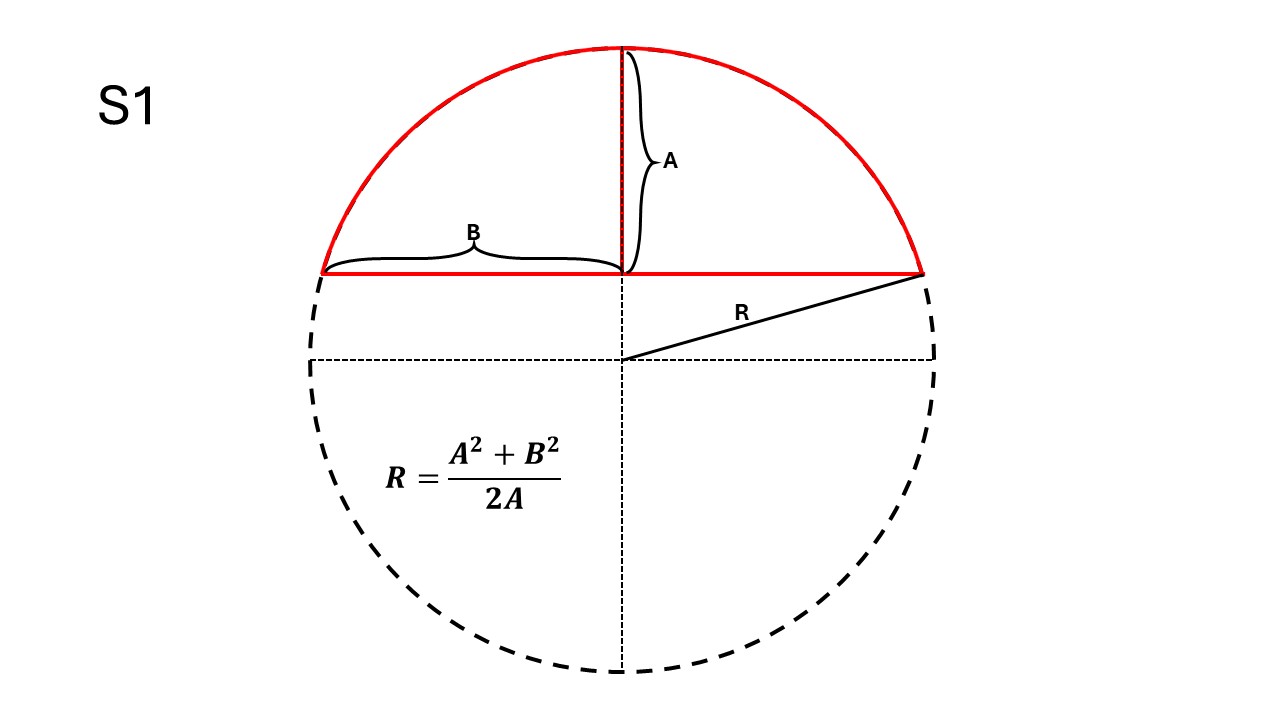


**Figure S1.** Method for calculating the bending radius in flexible tests. Here A represents the maximum height of the bend, while B represents half of the lateral width of the bent substrate, and R represents the radius of the bend. Denoted in red is the physical region of the bent chips, while the dashed lines correspond to the theoretical circle being created by bending the chips.

$\epsilon= \frac{\tau}{R}$ (1)

**S2. Device Variation: Thickness, Resistance, Size & Placement**

Thickness of the devices were tested using optical profilometry, creating both 2D maps of device height (Fig. S2-1.a) and individual data per device. Individual heights are matched with device performance and then compared across entire arrays (Fig. S2-1.b), following this methodology no relationship between thickness and device performance can be extracted. This result is not unexpected as thickness measurements show that all devices are beyond 10 layers thick, falling firmly within the bulk range for MoS­_2_ material characteristics and seeing only slight changes based on thickness. Much of the difference can likely be attributed to inherent resistive differences between flakes, and from overall active regions of devices which are dependent on the individual flake size.


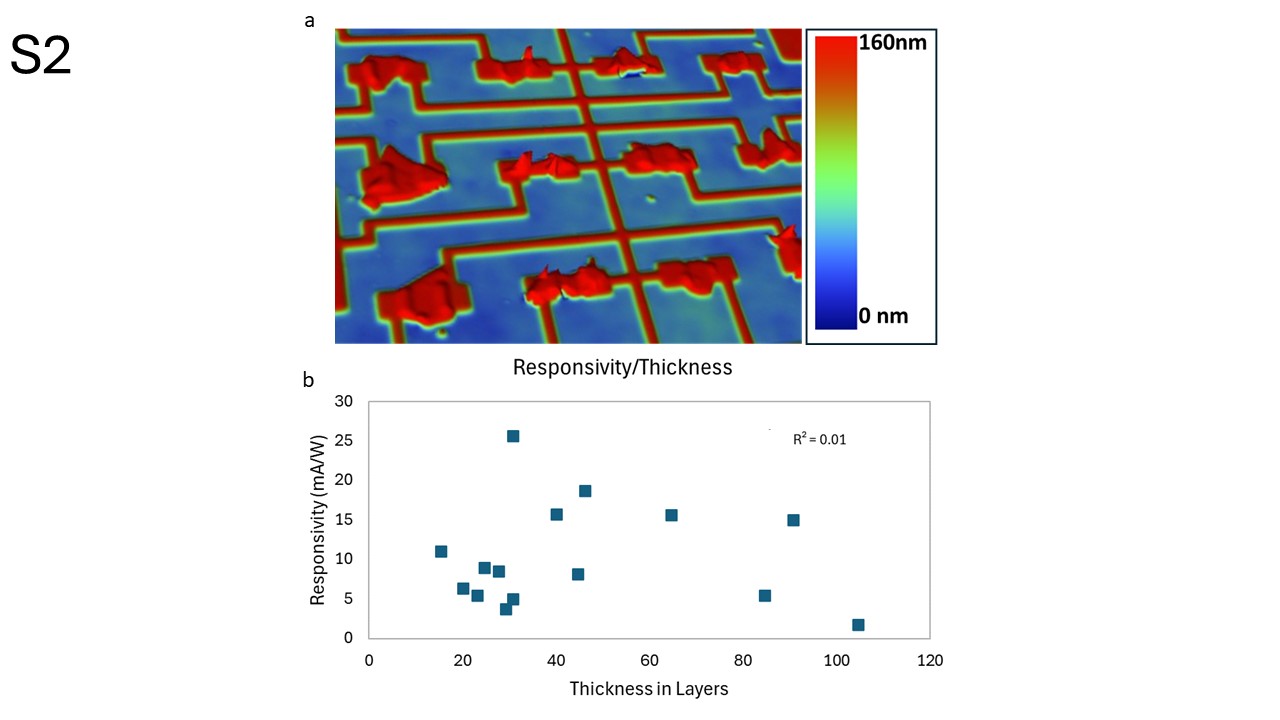


**Figure S2-1.** Thickness of devices & Analysis of thickness. **a,** An example of a 2D mapping of thickness from optical profilometry, on a 50 μm pitched array on a PI substrate, here devices have a maximum thickness of 160 nm. Also shown here is a non-uniformity in substrate flatness, this comes from the manufacturing process of the polyimide material and is typical across polymer sheets. **b,** Analysis of a photodetector array by matching the thickness of each device and its device performance and then comparing against all devices in the array. Following this methodology the best fit shows and R^2^ value of only 0.01 indicating there is no correlation between the thickness and device performance.

A power correlation can be drawn to the resistance of the devices and their response, however here we find two different groups of devices (blue and orange data, **Fig. S2-2**). One of those groups has a higher response per resistance than the other leading to the conclusion that another effect is occurring that is also contributing to the device variation. One explanation for devices 11, 12, and 14 showing a higher photo responsivity, is that the size and placement of these devices is smaller than that of others flakes and fit ‘cleanly’ between the two electrodes. Such an alignment could allow for greater collection of photo-generated carriers, in contrast to other devices where the 2D flake size supersedes the area of the electronic lead pads; i.e. those devices are not as cleanly placed on the electrodes creating a more difficult extraction path (higher resistance) for the short carrier lifetimes (e.g. device 3). Thus, leading to the conclusion that material resistance, as well as size & placement of the devices on the electrode array led to the device variations. Finally, it is possible that absorption of individual devices could vary and, as has been shown in other publications, trapping of photogenerated carriers could occur due to the substrate-to-2D material interface [1], both of which could contribute to variation between devices. Passivation and interface engineering, e.g. by utilizing hBN atop and underneath the 2D material channel, enables a reduction to the device variation.

**References:**


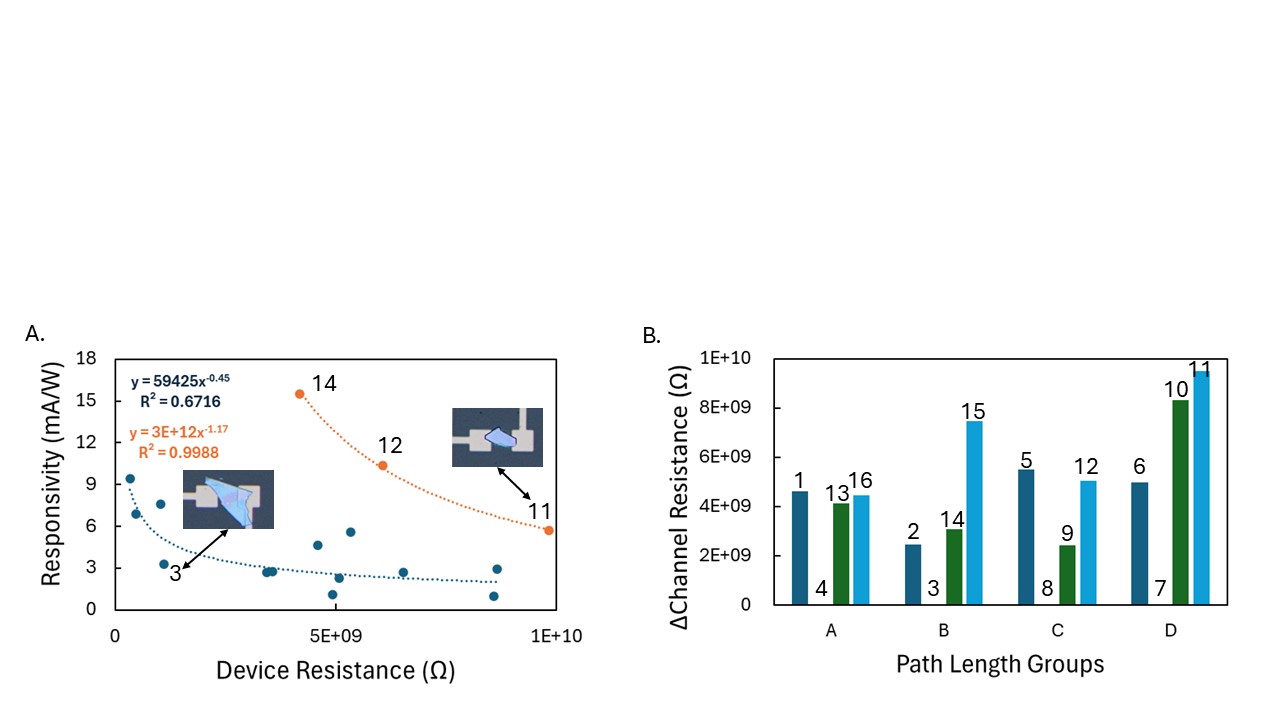


**Figure S2-2.** Device resistance analysis. **A.** Device resistance compared with responsivity; two clear trends emerge both in the form of exponential trends whereas the total device resistance increases the response decreases. This general trend makes physical sense, since resistance is increasing the photo-generated carriers will be more difficult to be collected. Separation of the two trends could be described by the device size and electrode placement. Here we point out also the higher response group devices, 11, 12, & 14, as well as device 3 for comparison for size and placement. **B.** Comparison of channel resistance, where the contact and electrical path resistance has been accounted for using the symmetrical design of the array. Path length groups refer to the four devices in the array which share the same configuration for electrical path, thus leading to the changes between to be described as the difference in channel resistance. Here we find devices 3, 4, 7, and 8 to have the lowest channel resistances corresponding to the highest dark currents. Each device is labelled with in its group and then individual devices are shown above their respective resistances. Path lengths A, B, C, & D, refer to 320, 200, 270, & 230 μm respectively. This analysis assumes that Au-to-MoS_2_ contact resistance is static or varies to a significantly smaller degree than channel resistance for all devices.

[1] Minh Dao Tran, J. Kim, H. Kim, M. Doan, Dinh Loc Duong, and Young Hee Lee, “Role of Hole Trap Sites in MoS2 for Inconsistency in Optical and Electrical Phenomena,” ACS Applied Materials & Interfaces, vol. 10, no. 12, pp. 10580–10586, Mar. 2018, doi: https://doi.org/10.1021/acsami.8b00541.

**S3. Fabrication Procedures**

One main fabrication procedure was used to fabricate arrays on both SiO_2_ and PI substrates, with slight alterations to allow for electron beam lithography on the polymer substrate. The patterning was done using the Raith Pioneer e-beam lithography machine using PMMA as the resist, with extra coating of a water-soluble conductive polymer used on PI substrates. Evaporation was performed for the metallization step using a CHA Criterion electron beam evaporator, depositing a metal stack of consisting of 5 nm of titanium followed by 45 nm of gold, this recipe was used for all substrates. 2D material transfer was done using the system outlined in the main text directly transferring each device one at a time. Once arrays were complete, they were then placed in a strong vacuum to ensure adhesion and finally annealed in an inert environment following procedures listed in the main text.


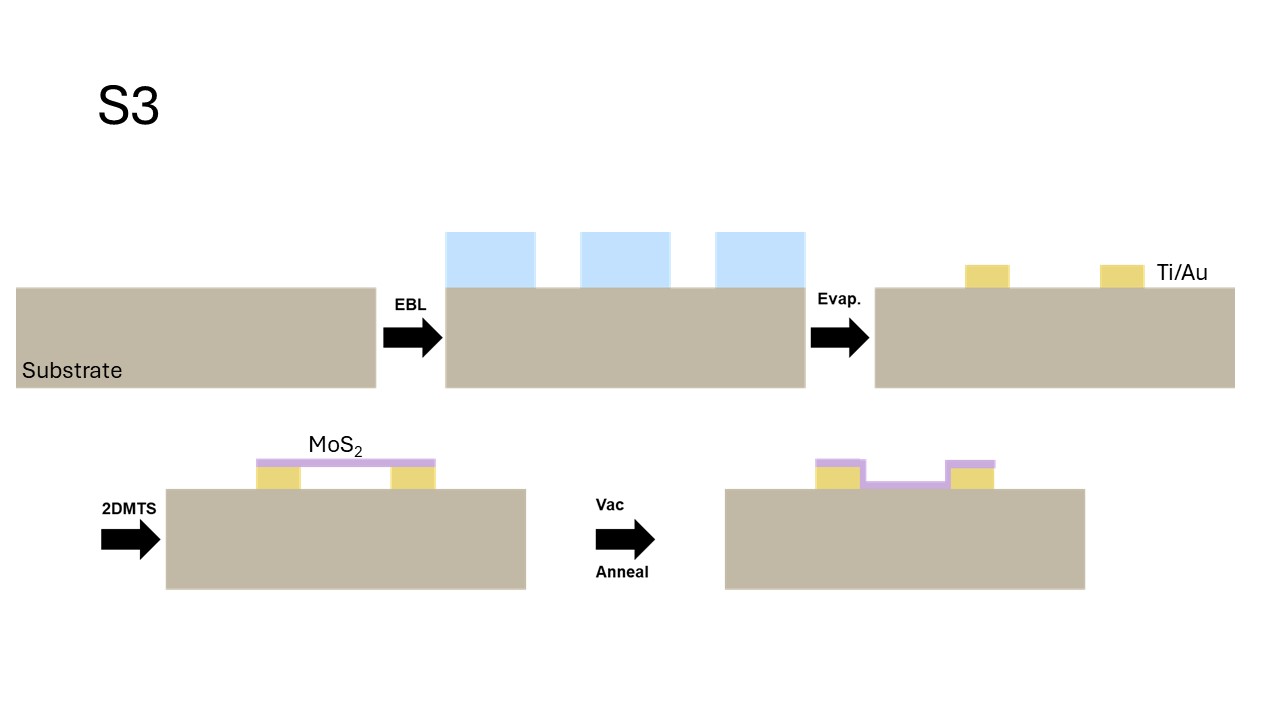


**Figure S3.** Fabrication procedure for devices. Once the substrate has been chosen the first step is using e-beam lithography to create the electrode design for the arrays, this was done with a positive resist and a conductive coating for fabrication on polymer substrates. With electrode patterns complete the metallization was done with an evaporation step creating a 5nm/45nm Ti/Au layer and then subsequently lifted off with special care taken not to warp polymer substrates in the process. Next the 2DMTS was used to place MoS_2_ flakes on the pre-patterned electrodes as outlined in the main text. Finally, the arrays were placed under a strong vacuum for at least two hours to ensure good adhesion of the MoS_2_ and then the final thermal annealing step was done in an inert environment before testing occurred.

**S4. Dark Current Changes under Bias Voltage Tuning**

In addition to characterizing and tuning the bias voltages to standardize the response of an array we have also investigated the effects of that tuning on the device dark currents. The variation in responsivity used by the bias tuning is based on the variable on/off ratio that can be seen in single devices performance when sweeping bias voltage, thus it follows that the same bias voltage tuning could have a significant effect on the dark current. To investigate this first the dark currents are characterized in the non-tuned state and then applied with tuning voltages and recharacterized. Analysis is then done by identifying the maximum and minimum dark currents across all devices in both operating conditions, those two values are then used as maxima and minima to linearly bin device performance into 20 different levels. Through this analysis method we identify a hot spot in the standard operating condition, where four devices are operating at significantly higher dark currents than the other devices, with a maximum of 5.9 nA. Additionally, we find that there are 4 more devices outside the hotspot regime that exist in a higher bin than the most populous bin. Overall, the analysis of the standard operating conditions show that the array has devices in 5 different bin levels, a maximum spread of 5.67 nA, and bin populations as follows: 50%, 25%, 12.5%, 6.25%, and 6.25%. The same process is then applied to the tuned system, which upon first inspection shows a significant diminishing of the hotspot, with two of the four devices no longer contributing to hotspot and the other two showing a significant decrease in the dark current level. The overall analysis in the tuned operating conditions show devices in 3 different bin levels, a maximum spread of 2.76 nA, and bin populations as follows: 87.5%, 6.25%, and 6.25%. This analysis shows that the tuning used for consistent responsivities is also working towards providing consistent dark currents, although there is still some disagreement in the dark current while the tuning voltages provide full agreement in the responsivity. This result is consistent with the methodology applied to the tuning voltages, where most of the devices have been tuned down to lower bias voltages to reduce the on/off ratio. However, the two devices that still see higher dark currents had much lower reduction in the dark (off) current than in the on state, resulting in higher dark currents after tuning. It is also possible to set the bias tuning voltages such that all dark currents would be consistent, however this would come at the cost of consistency in the responsivities and a requirement for higher precision voltage control for the tuning (beyond the 100-mV used). Such a tuning would change device 7 & 8 by changing the bias tuning to -0.96 V & -0.92 V resulting in responsivities of 0.27 mA/W & 0.15 mA/W, respectively.


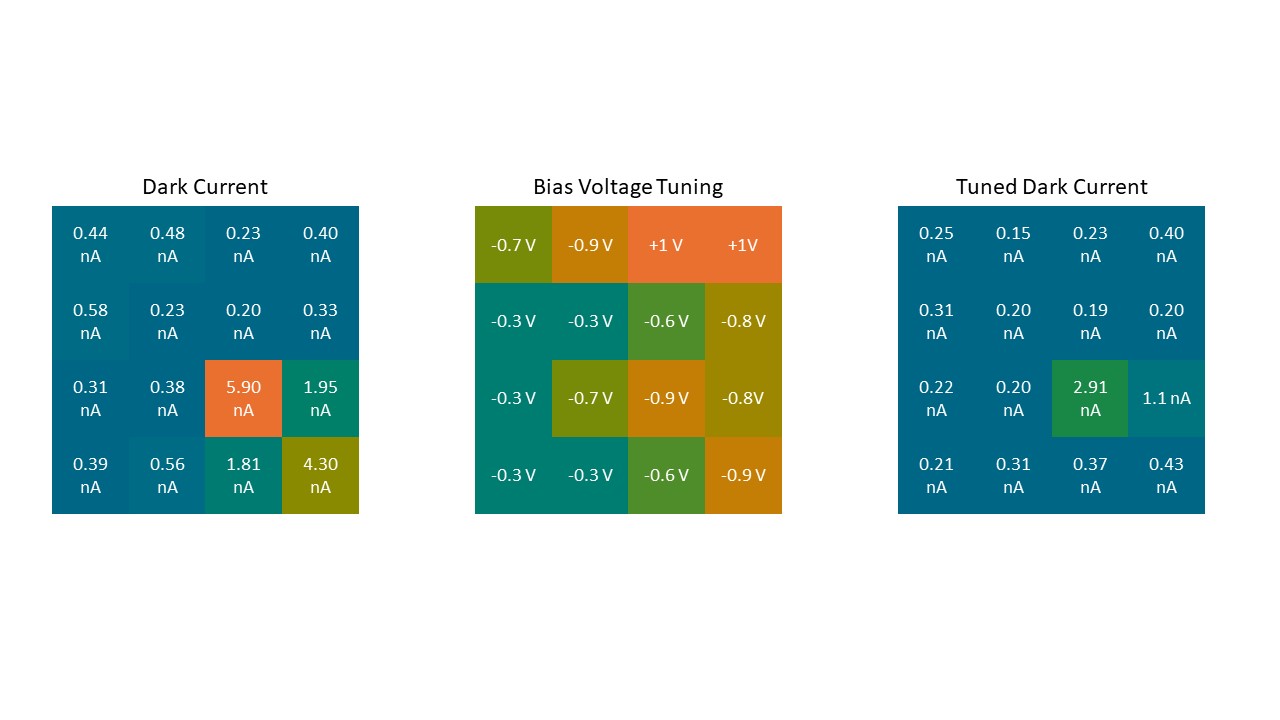


**Figure S4.** Bias voltage tuning effect on dark currents. Left side shows the dark currents before tuning showing a significant hotspot in the lower right corner of the array with four different devices showing dark currents above 1 nA and two of them showing dark currents above 4 nA, with a maximum of 5.9 nA. In addition to the four devices in the hotspot, four other devices show minimally increased currents, corresponding to higher bin when the range of dark currents was discretized to 20 levels. The devices were then subjected to the same adjustments to bias voltage tuning from a standardized 1 V bias as presented in the main text. The dark currents after tuning can be seen on the right side of the figure, the distinct hotspot that existed before tuning has been diminished with two of the four devices now showing low dark currents consistent with the minimal bin level. The additional two devices with higher remaining dark currents have both been decreased with the new maximum at 2.91 nA, Additionally, the four devices outside of the hot spot which had slightly higher dark now sit firmly in minimal bin level.
